# Complete Genome Sequences of Nine Enterovirus D68 Strains from Patients of the Lower Hudson Valley, New York, 2016

**DOI:** 10.1128/genomeA.01394-16

**Published:** 2016-12-15

**Authors:** Weihua Huang, Changhong Yin, Jian Zhuge, Taliya Farooq, Esther C. Yoon, Sheila M. Nolan, Donald Chen, John T. Fallon, Guiqing Wang

**Affiliations:** a Department of Pathology, New York Medical College, Valhalla, New York, USA; b Department of Pathology and Clinical Laboratories, Westchester Medical Center, Valhalla, New York, USA; c Division of Pediatric Infectious Disease, Department of Pediatrics, New York Medical College, Valhalla, New York, USA; d Department of Medicine, New York Medical College, Valhalla, New York, USA

## Abstract

Complete genome sequences of nine enterovirus D68 (EV-D68) strains from patients in New York were obtained in 2016 by metagenomic next-generation sequencing. Comparative genomic analysis suggests that a new subclade B3, with ~4.5% nucleotide divergence from subclade B1 strains causing the 2014 outbreak, is circulating in the United States in 2016.

## GENOME ANNOUNCEMENT

Enteroviruses in the family *Picornaviridae* are small, nonenveloped viruses with a single-stranded, positive-sense RNA genome of approximately 7.5 kb. The genus *Enterovirus* contains seven species, including enterovirus A to D and rhinovirus A to C that commonly cause human disease. Enterovirus D68 (EV-D68) was first recovered from patients with respiratory illness in California in 1962 (1) and was infrequently reported until its recent emergence worldwide (2). In August 2014, a nationwide outbreak of EV-D68-associated severe respiratory illness with at least 1,153 confirmed cases was reported from 49 U.S. states (3, 4). There were no confirmed cases in 2015 and only limited sporadic cases in 2016 in the United States (4).

Westchester Medical Center is a tertiary healthcare facility with a children’s hospital mainly serving patients in the lower Hudson Valley, New York. During the 2014 U.S. outbreak, we identified 95 children with EV-D68 infection from September and October 2014 using an EV-D68-specific real-time reverse transcription-PCR (rRT-PCR) (5) and a shotgun next-generation sequencing assay (6). In contrast, none of the 186 nasopharyngeal (NP) swab specimens from our patients from September and October 2015 were positive for EV-D68.

From 1 June to 30 September 2016, 358 of 1,219 (29.4%) NP specimens were positive for rhinovirus/enterovirus (RhV/EV) by the FilmArray Respiratory Panel (BioFire, Salt Lake City, UT, USA). EV-D68 was detected by rRT-PCR in 125 of 346 (36.1%) RhV/EV-positive NP specimens from 114 children and 11 adults, ages ranging from 4 weeks to 90 years (median = 3 years). For the 104 pediatric patients with clinical data available, 31 (29.8%) patients required pediatric intensive care in 2016.

Complete genome sequences of EV-D68 strains from 9 of our patients in 2016 were obtained by shotgun metagenomic next-generation sequencing using the MiSeq system (Illumina, San Diego, CA, USA) as described previously (7), with the exception that paired-end sequencing (2 × 150 bp) was performed. Raw read sequences were aligned and curated using a reference genome (strain NY120, accession no. KP745751) from a 2014 patient.

Comparative genome analysis of 325 EV-D68 strains available from GenBank, including nine from this study and three from patients with acute flaccid myelitis (AFM) as reported by the CDC (accession nos. KX675261 to KX675263), suggest that a novel subclade B3 of EV-D68 (8) is circulating in the United States in 2016, with ~4.5% nucleotide divergence from subclade B1 strains causing the 2014 outbreak.

It is unclear to date if our observation indicates local or nationwide activity of EV-D68 in 2016. With an increasing number of AFM cases reported, clinicians and public health agencies should be aware of the active circulation of EV-D68 and its clinical implications.

### Accession number(s).

The complete genome sequences of nine EV-D68 strains from the present study have been deposited in GenBank under the accession numbers KX957754 to KX957762.
